# Autophagy and mitochondrial remodelling in mouse mesenchymal stromal cells challenged with *Staphylococcus epidermidis*

**DOI:** 10.1111/jcmm.12518

**Published:** 2015-02-26

**Authors:** Nikolai V Gorbunov, Dennis P McDaniel, Min Zhai, Pei-Jyun Liao, Bradley R Garrison, Juliann G Kiang

**Affiliations:** a Radiation Combined Injury Program, Armed Forces Radiobiology Research InstituteBethesda, MD, USA; b Biomedical Instrumentation Center, Uniformed Services University of the Health SciencesBethesda, MD, USA; c Department of Radiation Biology, Uniformed Services University of the Health SciencesBethesda, MD, USA; d Department of Medicine, Uniformed Services University of the Health SciencesBethesda, MD, USA

**Keywords:** mesenchymal stromal cells, *Staphylococcus epidermidis*, stress response, autophagy, mitochondria

## Abstract

The bone marrow stroma constitutes the marrow-blood barrier, which sustains immunochemical homoeostasis and protection of the haematopoietic tissue in sequelae of systemic bacterial infections. Under these conditions, the bone marrow stromal cells affected by circulating bacterial pathogens shall elicit the adaptive stress-response mechanisms to maintain integrity of the barrier. The objective of this communication was to demonstrate (*i*) that *in vitro* challenge of mesenchymal stromal cells, *i.e*. colony-forming unit fibroblasts (CFU-F), with *Staphylococcus epidermidis* can activate the autophagy pathway to execute antibacterial defence response, and (*ii*) that homoeostatic shift because of the bacteria-induced stress includes the mitochondrial remodelling and sequestration of compromised organelles *via* mitophagy. Implication of Drp1 and PINK1–PARK2-dependent mechanisms in the mitophagy turnover of the aberrant mitochondria in mesenchymal stromal cells is investigated and discussed.

## Introduction

Immunosuppression and impairment of immune barriers often result in translocation of resident microorganisms such as Gram-positive *Staphylococcus epidermidis*, which can appear in the peripheral blood and then in vital organs and tissues 1. In bone marrow, the immune homoeostasis and defence response to blood pathogens are mediated by the marrow-blood barrier, which is comprised of endothelial, reticuloendothelial and mesenchymal stromal cell lineages 2–5. *In vivo* assessment of bacterial effects on the marrow-blood barrier supported by these cell lineages is a difficult task because of the complexity of spatial–temporal interactions of the cells in the tissue 2,4. However, it is reasonable to assume that the stress responses originating from each of the stromal cell lineages experiencing bacterial impact are integrated in the stromal tissue to form a general defence response of the ‘barrier singularity’. Therefore, the results of *in vitro* differential assessment of antibacterial responses of the stromal cell lineages might be further ‘translated’ for picturing events initiated in the stromal tissue 6.

We recently reported that bone marrow mesenchymal stromal cells (BMSCs) challenged *in vitro* with Gram-negative *Escherichia coli* can up-regulate autolysosomal machinery decomposing the phagocytized microorganisms 6. These events are accompanied by activation of a battery of stress-response mechanisms, which makes BMSCs resistant to the bacterial pathogens 6. Effects of a Gram-positive organism on BMSCs are poorly investigated. The focus of the study presented in this paper was towards elucidation of the role of macroautophagy (thereafter, *autophagy*) in antibacterial defence response and mitochondrial remodelling associated with the pathogen-induced stress in murine clonal BMSCs challenged with Gram-positive *S. epidermidis, i.e*. a species commonly present on skin and easily penetrating into the internal body through wounds.

The autophagy–autolysosomal pathway is considered to be an evolutionarily developed pro-survival mechanism, which is essential for removal of unnecessary cellular constituents and pathogens 7–11. Although autophagy has been originally defined as a non-specific degradation process, one line of evidence suggests that some autophagy pathways (such as sequestration of invading bacteria and autophagy of damaged organelles) selectively execute quality control of the cell environment 11,12. We hypothesized that a challenge of BMSCs with *S. epidermidis* can induce a set of complex pro-survival responses that include the autophagy-selective degradation of the phagocytized microorganisms (*i.e*. xenophagy), remodelling of mitochondrial network *via* fission/fusion mechanisms, and autophagy of aberrant mitochondria (*i.e*. mitophagy).

While the detailed mechanisms driving selective autophagy at the site of microbial phagocytosis remain under investigation, it is broadly accepted that activation of the pattern recognition elements (PREs), the damage-associated molecular patterns (DAMPs), such as high-mobility group protein B1 (HMGB1) and immunity-related GTPase family M protein (IRGM) can trigger xenophagy events and cascades of adaptive responses mediated by transcriptional stress factors 6,11,13–16. The initial autophagy step is defined as induction of phagophores and their elongation to autophagosomal membranes. These events proceed *via* a cascade mechanism, which requires activation of (*i*) the Ulk1–Atg13–FIP200 kinase complex, (*ii*) the autophagy-specific class III PI3-kinase/Vps34/p150/Beclin 1 complex (I), (*iii*) the PI3P-binding Atg2/Atg18 complex, (*iv*) protein kinase D (PKD), a redox-sensitive activator of Vps34 and (*v*) the two ubiquitin-like conjugation systems: Atg5-Atg12/Atg16L1, and ATG8/LC3 (MAP1). The associated recognition of ubiquitinated bacterial cargo and its enclosure into autophagosome occurs *via* adaptor/cargo-receptor proteins (such as p62/SQSTM) followed by autophagosomal fusion with lysosomes and further maturation mediated by Vps34/Beclin 1/UVRAG complex (II) 11–14,17. The sequestered bacterial cargo is decomposed within autolysosomes.

Surprisingly, numerous data interconnect the cell antibacterial mechanisms mediated by PREs, DAMPs, IRGM and autophagy with the pathogen/inflammagen-induced mitochondrial remodelling 6,11,13,15–17. In this light, interplay between xenophagy and mitophagy may play the crucial role in the cell defence response 7,8,11,13,15–20. Thus, it has been suggested that mitochondrial fission and mitophagy of the compromised organelles are pre-requisites for successful cell survival 7,8,18,19. In these events, mitochondrial fission plays a crucial role in (*i*) segregation of aberrant mitochondrial fragments from the rest of tubular network and (*ii*) providing membrane sources for autophagosomal biogenesis 7,8,18,21–24. Mitochondrial fission is regulated by Drp1 and Fis1 proteins, which are predominantly localized in cytoplasm and on the mitochondrial outer membrane (MOM), respectively 23,25. The interaction between Drp1 and Fis1 and subsequent mitochondrial fission occurs when post-translationally modified Drp1 protein is recruited to MOM 22,23,26. A priming of damaged mitochondria for mitophagy can implicate NIX, BNIP3, FUNDC1 or PINK1 (*i.e*. MOM-located PTEN-induced putative kinase 1) and PARK2 (*i.e*. E3-ubiquitin ligase Parkin)-dependent mechanisms 9. In the PINK1/PARK2-dependent mechanism, mitochondrial damage and depolarization triggers: (*i*) impaired stabilization of PINK1 in mitochondria; (*ii*) PINK1-dependent recruitment of PARK2 to mitochondria and (*iii*) PARK2-dependent poly-ubiquitination of proteins (*e.g*. voltage-dependent anion channel VDAC1) 9,17,24,27,28. The poly-ubiquitinated mitochondrial proteins are then recognized by the autophagic adapter protein SQSTM1/p62, which has a structural motif (WXXL) compatible with LC3 II, a target protein on nascent autophagosomal membrane 11,12,29,30. Eventually, this sequence of events results in autophagosomal enclosure of the impaired mitochondria mediated by the SQSTM1/p62-LC3 II-cargo-receptor complex; while the cargo-clearance occurs after autophagosomal fusion with lysosomes and further maturation mediated by Vps34/Beclin 1/UVRAG complex (II) 7,11,12,24,30.

The recently well-described proteins involved in mitochondrial fusion events are mitofusins (Mfn1 and Mfn2) and optic atrophy protein 1 (Opa1) 19,21–23. Mnf1 and Mnf2 are outer mitochondrial membrane proteins that mediate fusion through their cytoplasm-exposed GTPase domains allowing adhesion and fusion of outer mitochondrial membranes. Opa1 is an inner mitochondrial membrane protein, which subsequently enables fusion of inner mitochondrial membranes 19,21,22. This fusion mechanism promotes formation of long tubular mitochondria with their further interconnection into reticular structures extending in three dimensions over the cell volume 19.

Overall, according to recent paradigm, the pathogen-induced mitochondrial remodelling can be considered as a part of the global cell signalling system, which orchestrates defence responses to danger and injury 9,10,17,18,20,31–33.

In this report, we provide evidence that BMSCs challenged with *S. epidermidis* can activate a complex of antibacterial defence mechanisms and stress responses including up-regulation of phagocytosis and the autophagy/autolysosomal machinery. These events were accompanied by structural alterations in the mitochondrial network and by increases in (*i*) translocation of Drp1 and PARK2 protein towards mitochondria, (*ii*) interaction of the mitophagy-mediated proteins, such as SQSTM1/p62, and LC3, with mitochondria and (*iii*) biodegradation of aberrant mitochondria *via* the mitophagy pathway.

## Materials and methods

### Mouse BMSCs

The cultures of bone marrow stromal cells (CFU-F) were established and expanded as described previously 6. Briefly, bone marrow was obtained from 3- to 4-month-old B6D2F1/J female mice using a protocol adapted from STEMCELL Technologies, Inc. (www.stemcell.com/~/media/Technical%20Resources/0/0/29018_Mesenchymal.pdf).

The mesenchymal stromal cells were expanded and cultivated in hypoxic conditions (5% O_2_, 10% CO_2_, 85% N_2_) for approximately 30 days in Mesencult medium (STEMCELL Technologies, Inc.) in the presence of antibiotics. After five passages and formation of BMSC colonies (Supplement A), three selected colonies were collected, and trypsinized; then the cell suspensions (approximately 5 cells/ml) were aliquoted in three 96-well plates (0.1 ml/well) for cloning as described previously 34. After 2-week cultivation of the cells in the plates, a clone from a selected well was collected and expanded during another 2 weeks 34. The phenotype of the obtained clonal BMSCs (Supplement B) was assessed with flow cytometry and immunofluorescence imaging using positive and negative markers for mesenchymal stromal cells as suggested in (www.rdsystems.com/Products/SC018) (see below).

### Phenotype assessment of clonal BMSCs

Phenotyping of the cultured cells was conducted using immunofluorescence labelling of cell surface proteins with antibodies against conventional positive markers of BMSCs (*i.e*. Sca-1, CD44 and CD105) and negative markers (*i.e*. CD4, CD34 and myeloperoxidase). That was followed by either flow cytometry or fluorescence microscopy analysis (see Materials and methods below).

For flow cytometric analysis, the harvested cells (three specimens per group) were re-suspended in Hank's Balanced Salt Solution containing 10% foetal bovine serum using polypropylene tubes. Then, the cells were incubated either with rat IgG-biotin against mouse CD44 (BioLegend, San Diego, CA, USA), or with rat IgG-biotin against mouse Ly-6A/E (Sca-1) (BioLegend), or with rat IgG against mouse CD4 conjugated with Pacific Blue™ (BioLegend), or mouse IgG against CD34 conjugated with Alexa Fluor® 647 (eBioscience, San Diego, CA, USA), or rat IgG-biotin against mouse CD117 (eBioscience) for 20 min. at room temperature. Isotype controls were mouse IgG conjugated with Alexa Fluor® 647 (BioLegend), rat IgG conjugated with Pacific Blue™ (BioLegend), and rat IgG-biotin (BioLegend). Biotin counter-conjugates were streptavidin-Pacific Blue™ conjugate and streptavidin- Alexa Fluor®647 conjugate from Life Technologies Inc., Grand Island, NY, USA. Positive marker-specific immunofluorescence of BMSCs was distinguished from the isotype immunofluorescence (negative control for non-specific immunoadherence) in all samples. The cells were analysed with the BD™ LSRII Flow Cytometer (BD Biosciences Co., www.bdbiosciences.com). The histogram data represent cell counts per 10,000 events. The figures are shown in Supplements C-H.

### Challenge of BMSCs with bacteria

Bone marrow mesenchymal stromal cells cultures (approximately 90% confluency) were challenged with *S. epidermidis* (5 × 10^7^ bacteria/ml) for 3 hrs in antibiotic-free Mesencult MSC Medium (STEMCELL Technologies, Inc.) (Supplement I). Then, the incubation media was replaced with fresh medium containing penicillin and streptavidin antibiotics, and BMSCs were further incubated until cell collection 6. The cells were harvested at 3, 5 and 24 hrs following bacterial challenge. The cells displayed remarkable resistance to the bacterial challenge and sustained confluency over the period of observation (Supplement J). For interference with the autophagosomal/lysosomal fusion promoted by the bacterial challenge, BMSCs were either pre-incubated with 50 μM vinblastine (Cat. # V1377; Sigma-Aldrich Corp., St. Louis, MO, USA) for 6 hrs before fixation, or transfected with siRNA targeting mouse Rab 7 (Cat. # 4390771; Life Technologies, Inc. Grand Island, NY, USA) using Lipofectamine®RNAiMAX Transfection Reagent (Cat. # 13778030; Life Technologies, Inc. Grand Island, NY, USA) as per the manufacturer's instructions (www.lifetechnologies.com/order/catalog/product/13778030). Inhibition of cell signalling pathways downstream of NFκB and PI3K/AKT activated by pattern recognition receptors in the challenged BMSCs was produced in the presence of 10 μM pyrrolidine dithiocarbamate (PDTC; Cat. P8765; Sigma-Aldrich Corp., St. Louis, MO, USA) and 200 nM Wortmannin (Cat. #9951; Cell Signaling Technology Inc.). L-N^6^ -(1-iminoethyl)lysine (LNIL, Cat. #I8021; Sigma-Aldrich Corp., St. Louis, MO, USA), a selective inhibitor of iNOS, was used for suppression of nitric oxide production in the cells. To interfere with post-transcriptional synthesis, the cells were pre-incubated with 10 μM 5-Azacytidine (5-AzaC) which is incorporated into RNA disrupting nucleic acid and protein metabolism (Cat. # A2385; Sigma-Aldrich Corp., St. Louis, MO, USA.).

Bone marrow mesenchymal stromal cells were analysed for (*i*) viability, pro-apoptotic alterations, integrity of cell monolayers and colony-forming activity; (*ii*) bacterial phagocytosis and autophagy; (*iii*) response of stress proteins and (*iv*) remodelling of mitochondrial network and mitophagy, using quantitative real-time PCR technique (qRT-PCR), fluorescence confocal imaging, protein immunoblotting, bright-field microscopy and transmission electron microscopy (TEM).

The apoptotic response in BMSCs was determined by immunoblot analysis of caspase-3, a marker of apoptosis.

The images presented in Supplements I-J indicate that the BMSC cultures challenged with bacteria were also able to sustain integrity of confluent monolayers; there were no signs of pro-apoptotic alterations in BMSCs (see below).

### BMSC protein expression analysis using qRT-PCR

Up-regulation of gene expression of was assessed with the qRT-PCR technique. BMSCs were collected at 3, 5 and 24 hrs following bacterial challenge and subsequently subjected to lysis. Total cellular RNA was isolated from the cell lysates using the Qiagen RNeasy miniprep kit (Cat. # 74104; Qiagen Co.), quantified by measuring the absorbance at 260 nm, and qualified by electrophoresis on a 1.2% agarose gel. cDNA was synthesized using Superscript II (Cat. #18064-014; Invitrogen™) and qRT-PCR was performed with SYBR Green iQ Supermix (Cat. # 170-8880; Bio-Rad Laboratories, Inc.), each according to the manufacturers’ instructions. A set of primers used for qRT-PCR is presented in Table1; the primers were purchased from Integrated DNA Technologies, Inc. (www.idtdna.com). The quality of qRT-PCR data were verified by melt curve analysis, efficiency determination and agarose gel electrophoresis. Relative gene expression was calculated by the method of Pfaffl using the formula 2^−ΔΔCt^.

**Table 1:** A list of primers applied for assessment of transcriptional gene activation in BMSCs challenged with *Staphylococcus epidermidis*

Gene	Forward	Reverse
*IRGM*	GAGAGAGAGAGCAGGGCAC	AGCATAATGGGTCTCTGCCA
*NOS2*	CAGCTGGGCTGTACAAACCTT	CATTGGAAGTGAAGCGTTTCG
*IL1*α	CGGGTGACAGTATCAGCAAC	GACAAACTTCTGCCTGACGA
*p65-NFkB*	GCGGCCAAGCTTAAGATCTGC	CGCTGCTCTAGAGAACACAATG
*Atg5*	CCTGAAGATGGAGAGAAGAG	GGACAATGCTAATATGAAGAAAG
*Atg8*	AGGCCATETTCCTGTTTGTG	GTGTTCTCTCCGCTGTAGGC
*Mfn1*	ACAAGCTTGCTGTCATTGGG	GCATTGCATTGATGACAGAGC
*PARK2*	GAGCTTCCGAATCACCTGAC	CCCTCCAGATGCATTTGTTT
*Pink1*	TGCCTGAGATGCCTGAGTC	GCAGCACATTTGCAGCTAAG
*p62/SQSTM1*	CGAGTGGCTGTGCTGTTC	AGCCATTGTCAGCTCCTCAT

### Analysis of the cell proteins

Proteins from BMSCs were extracted in accordance with the protocol described previously 6. Aliquots of proteins were resolved on SDS-polyacrylamide slab gels (NuPAGE 4–12% Bis-Tris; Cat.# NP0322BOX; Life Technologies, Inc.). After electrophoresis, proteins were blotted onto a PDVF membrane and the blots were incubated with antibodies (1 μg/ml) raised against MAP LC3 (Cat.# L8918; Sigma-Aldrich Corp., St. Louis, MO, USA), Lamp1 (Cat. # NB100-77683; Novus Biologicals, LLC) rat IgG, HMGB1 (Cat.# PAB12414; Abnova Co.), Drp1 (Cat.# 3455; Cell Signaling Technology, Inc. Beverly, MA, USA), PINK1 (Cat.# sc33796; Santa Cruz Biotechnology, Inc. Dallas, TX, USA), Park2 (Cat.#ab15954; Abcam Plc.), Sirt3a (Cat.#ab86671; Abcam Plc., Cambridge, MA, USA), SUMO1 (Cat.#4940; Cell Signaling Technology, Inc. Beverly, MA, USA), p62/SQSTM1 (Cat.# ab91526; Abcam Plc., Cambridge, MA, USA), Mfn1 (Cat.#sc50330; Santa Cruz Biotechnology, Inc. Dallas, TX, USA), IGRM (Cat. # LS-B2732; LifeSpan BioSciences, Inc., Seattle, WA, USA) rabbit IgG, Tom20 (Cat.# WH0009804M1; Sigma-Aldrich Corp., St. Louis, MO, USA), HSP70 (Cat.#sc33375; Santa Cruz Biotechnology, Inc. Dallas, TX, USA), Casp-3 (d.b.a. EMD Millipore, Cat. #04-439) and actin (Cat.# A5441; Sigma-Aldrich Corp., St. Louis, MO, USA) mouse IgG followed by incubation with species-specific IgG peroxidase conjugate. LC3I and LC3II proteins were identified using mouse brain protein extract (Cat.#78501; Thermo Fisher Scientific Inc., Danvers, MA, USA) as a positive control.

### Immunoprecipitation (IP)

Tissue lysates containing 300 μg protein were incubated with antibodies against Tom20 (*i.e*. translocase of outer mitochondrial membrane 20; Cat.#WH0009804M1; Sigma-Aldrich Co.), chilled on ice for 1 hr, mixed with protein A/G agarose beads (50 μl; Cat.#sc2003; Santa Cruz Biotechnology), and incubated overnight at 4°C. The immunoprecipitate was collected by centrifugation at 12,500 × g for 10 min., washed twice with 500 μl RIPA stop buffer (Cat.#R0278; Sigma-Aldrich Co.) and once with 500 μl PBS (Cat.#10010; Life Technologies Corporation) wash buffer. The pellet was re-suspended in 32.5 μl PBS (Cat.#10010; Life Technologies Corporation), 12.5 μl 4× LDS sample buffer (Cat.# NP0007; Life Technologies Corporation) and 5 μl Sample Reducing Agent (Cat.# NP0009; Life Technologies Corporation), boiled for 5 min. and then centrifuged for 30 sec. to remove the agarose beads. The supernatant containing mitochondrial proteins immunoprecipitated in complexes with Tom20 [such as PINK1 (Cat.# sc33796; Santa Cruz Biotechnology, Inc.) and PARK2 (Cat.#ab15954; Abcam Plc.)] were then analysed with immunoblotting, as described above. To test whether mitochondrial impairment up-regulates the interaction of PARK2 with mitochondria, and thus leads to increase in co-immunoprecipitation of PARK2 with mitochondrial PINK1, we induced the depolarization of mitochondrial membrane by incubation of BMSCs with 10 μM carbonyl cyanide 3-chlorophenylhydrazone (CCCP; Cat. #C2759; Sigma-Aldrich Co.), a mitochondrial uncoupler 15,23.

### Immunofluorescence techniques and image analysis

For immunofluorescence confocal imaging, BMSCs (five specimens per group) were grown on chambered coverglasses (Thermo Fisher Scientific, Inc.). BMSCs were fixed in 2% paraformaldehyde, processed for immunofluorescence analysis and analysed with fluorescence confocal microscopy 6. Normal donkey serum and antibody were diluted in PBS containing 0.5% BSA and 0.15% glycine. Any non-specific binding was blocked by incubating the samples with purified normal donkey serum (Cat.#2044; Santa Cruz Biotechnology, Inc.) diluted 1:20. Primary antibodies were against CD105 rat biotin-IgG (cat #120404; BioLegend, Inc.), myeloperoxidase (Cat.# sc-16128; Santa Cruz Biotechnology, Inc.) rabbit IgG, Tom 20 (Cat.# 56783; Abcam Plc., Cambridge, MA, USA) mouse IgG, Drp1 (Cat.# 3455; Cell Signaling Technology, Inc.), Park2 (Cat.# 15954; Abcam Plc.), Mfn1 (Cat.# 50330; Santa Cruz Biotechnology, Inc.), MAP LC3 (Cat.# L8918; Sigma-Aldrich Co.) rabbit IgG, IRGM (Cat. # LS-B2732; LifeSpan BioSciences, Inc.) rabbit IgG, SQSTM1/p62 (Cat.# ab91526; Abcam Plc) rabbit IgG, HMGB1 (Cat. #PAB18868; Abnova, Co.) rabbit IgG, Atg5 (Cat.# sc-8666; Santa Cruz Biotechnology, Inc.) goat IgG. That was followed by incubation with secondary fluorochrome-conjugated antibody and/or streptavidin-AlexaFluor 610 conjugate (Cat. #S32359; Molecular Probes, Inc.), and with Heochst 33258 (Cat.#H1398; Molecular Probes, Inc.) diluted 1:3000. Secondary antibodies used were Alexa Fluor 488® and Alexa Fluor® 594 conjugated donkey IgG (Cat.# A2106, A11058, A11055, A21207; Molecular Probes Inc.). Negative controls for non-specific binding included normal goat serum without primary antibody or with secondary antibody alone. Five confocal fluorescence images were captured with a Zeiss LSM 710 microscope (Carl Zeiss Microscopy GmbH, Jena, Germany). The immunofluorescence image analysis was conducted as described previously 6. The index of spatial correlation (R) of proteins was determined by multiple pixel analysis for pairwise signal interactions in green and red channels using Simple PCI High Performance Imaging software (Compix Inc., Cranberry Township, PA, USA) and ImageJ image analysis software (http://rsb.info.nih.gov).

Autophagic flux was determined by evaluating the punctuated pattern of MAP-LC3/Fluor 488® in the immunostained cells (punctae/cell were counted).

### Transmission electron microscopy (TEM)

Mesenchymal stromal cells in culture were fixed in 4% formaldehyde and 4% glutaraldehyde (catalog # 16000; Electron Microscopy Sciences, Hatfield, PA, USA) in PBS overnight, post-fixed in 2% osmium tetroxide (catalog # 19150; Electron Microscopy Sciences) in PBS, dehydrated in a graduated series of ethanol solutions, and embedded in Spurr's epoxy resin (catalog # 14300; Electron Microscopy Sciences). Blocks were processed as described previously 6. The sections of embedded specimens were analysed with a Philips CM100 electron microscope.

### Statistical analysis

The data were expressed as means ± SEM. Statistical significance was determined using Student's *t*-test (two-tailed) for independent samples. Significance was reported at a level of *P* < 0.05.

## Results

### Phagocytosis and autolysosomal degradation of *S. epidermidis* by BMSCs

Phagocytosis and autolysosomal degradation of *S. epidermidis* by BMSCs was analysed with TEM as recommended and published previously 6,29. The presence of high-contrast constituents in microorganisms can provide a unique opportunity for TEM observation of bacterial autophagy (or xenophagy) and biodegradation of autolysosomal contents.

**Figure 1:**
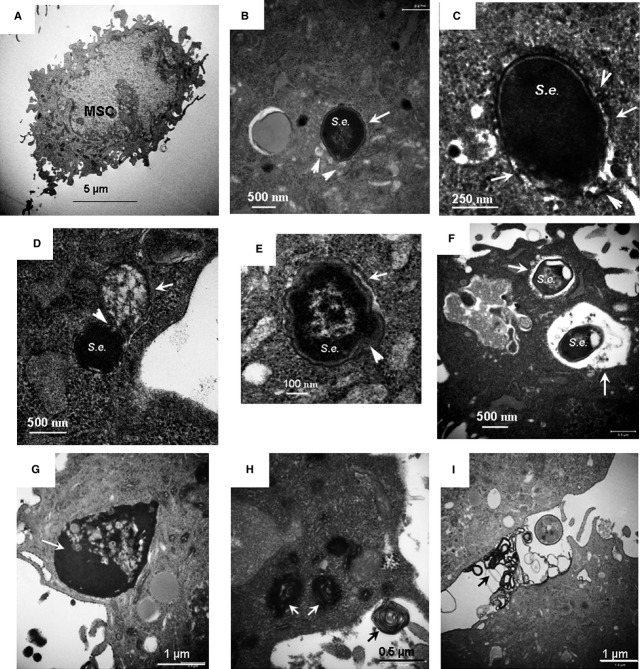
Transmission electron microscopy (TEM) analysis of phagocytosis and autophagy/autolysosomal processing of *Staphylococcus epidermidis* in BMSCs. (A) A micrograph of a control BMSC. (B and C) Micrographs of autophagosomal sequestration of *S. epidermidis* in BMSCs taken with different magnifications; BMSCs were fixed 5 hrs after the challenge. Autophagosome membranes are indicated with white arrows; fusion of small lysosomes with autophagosome encapsulating a microorganism is shown with white arrowheads. (D) Micrograph of fusion of autophagosome encapsulating a microorganism (white arrowheads) with a large autolysosome (white arrows); BMSCs were fixed 5 hrs after challenge. (E and F) Micrographs of degradation of sequestered *S. epidermidis* in large autolysosomes; BMSCs were fixed 24 hrs after challenge. (G–I) Formation of high-electron density secretory vacuoles in the challenged cells is indicated with white arrows; extracellular release of the secretory factors is indicated with black arrows. BMSCs were fixed 5 hrs after the challenge. Conditions: BMSCs were challenged with approximately 5 × 10^7^ bacteria/ml for 3 hrs. The cells were harvested and fixed for TEM 5 and 24 hrs after challenge.

Transmission electron microscopy images presented in Figure1 show a control cell (Fig.1A) and BMSCs subjected to bacterial challenge; the latter represents different stages of xenophagy and decomposition of microorganisms (Fig.1B–F). The events encompassed engulfing of the microorganisms into double-membrane autophagosomes (Fig.1B and C); the observed xenophagy was accompanied by fusion of autophagosomes with small lysosomes (20–30 nm in size; Fig.1B and C), indicating completion of autophagy flux. It should be noted that the assessment of autophagy flux was further conducted with immunofluorescence techniques and results of this assessment are discussed below. As shown in Figure1B and C, the autophagosomes engulfing the microorganisms were much larger (≈500 nm in size) than those formed during degradation of cellular organelles (*e.g*. mitochondria, ≈200 nm size, see below), suggesting that the elongation step of the autophagosome membrane is involved in the bacterial sequestration. The TEM image presented in Figure1D indicates that the autophagy-sequestered microorganisms can fuse with large autolysosomes (>500 nm). The following autolysosomal degradation of the sequestered microorganisms was characterized by fragmentation of bacterial constituents and abnormal notchings of bacterial shells (Fig.1E and F). As shown in the TEM micrographs (Fig.1G and H), the response of the cells to bacterial challenge was also accompanied by a massive formation of high-electron density secretory granules released into extracellular space (Fig.1I). Note, increase in secretory activity in the host cells may lead to the endoplasmic reticulum stress (ER stress) and/or activation of the ER–mitochondria axis in the host cells 35.

The observed activation of xenophagy was likely driven by stress/danger-responding mechanisms triggered by PREs. To address this assumption, we further analysed stress- and autophagy-related proteins in BMSCs challenged with *S. epidermidis*.

### Alterations in stress and autophagy-related proteins in BMSCs challenged with *S. epidermidis*

Recently, it has been demonstrated that *S. epidermidis*-derived inflammagens are potent in evoking numerous danger signals in the host phagocytic cells 36–38. Another line of evidence indicates that BMSCs display high levels of constitutively present adaptogens (*e.g*. HSP70, Sirt3), and can respond to bacterial inflammagens by induction and nuclear translocation of stress-response elements such as NFkB, TRX1, Ref1, Nrf2, FoxO3a, HO1, and activation of autophagy and mitochondrial remodelling 39. Therefore, it was reasonable to assume that the challenge of BMSCs with *S. epidermidis* could lead to up-regulation of a number of stress-responsive genes and mitophagy-related genes to sustain the cellular defence mechanisms.

A summary of quantitative assessments of these alterations is presented in Tables2 and 3. The challenge with *S. epidermidis* resulted in a significant transcriptional activation of the stress-responsive genes (such as *IRGM*, *NF*κ*B*, *NOS2*; Table2) and the mitophagy-related genes [such as *ATG5*, *ATG8* (MAP-LC3), *PARK2, PINK1, SQSTM1, Mfn1*; Table2]. Note, the transactivation of these genes in the host cells is often associated with pro-survival mechanisms mediated by the NFκB and PI3K/AKT pathways; while their suppression can be induced by inhibitors of the NFκB and PI3K/AKT responses, *e.g*. by PDTC and Wortmannin (individually or in combination).

**Table 2:** Assessment of transcriptional activation of stress-responsive genes in BMSCs challenged with *Staphylococcus epidermidis*

Genes	*S. epidermidis* challenge. Increase in expression (A.U.)	PDTC+Wortmannin treatment and *S. epidermidis* challenge. Increase in expression (A.U.)
*NFkB*	11.2 ± 3.9	1.9 ± 0.5 (^*^)
*NOS2*	1867 ± 909	28 ± 12 (^*^)
*IL1A*	238 ± 73	1.3 ± 0.4 (^*^)
*IRGM*	4.8 ± 0.9	0.32 ± 0.09 (^*^)

*S. epidermidis*-induced gene expression is presented as arbitrary units of relative expression over control ‘non-challenged’ group. The presented data are means ± S.E.M., *n* = 3. The gene expression data reported in the table are statistically significant with a confidence level of *P* < 0.05.

The statistical significance of inhibition of gene expression was assessed by using one-way anova with Tukey's *post hoc* HSD test. The observed differences between respective groups [*i.e*. inhibitors applied (*n* = 3) *versus* no inhibitors (*n* = 3)] were reported statistically significant (^*^) with a confidence level of *P* < 0.05.

**Table 3:** Assessment of transcriptional activation of autophagy-related genes in BMSCs challenged with *Staphylococcus epidermidis*

Genes	*S. epidermidis* challenge
*ATG5*	8.3 ± 3.7
*ATG8* (LC3)	7.4 ± 3.1
*Mfn1*	3.8 ± 1.4
*PARK2*	10.1 ± 6.1
*Pink1*	2.3 ± 0.4
*SQSTM1*	5.8 ± 2.2

The presented data are means ± SD, calculated from three independent experiments. We reported that expression response was statistically significant with a confidence level of *P* < 0.05.

Indeed, pre-incubation of BMSCs with a combination of 10 μM PDTC and 200 nM Wortmannin produced dramatic effects on the analysed stress-responsive genes. Thus, the drastic expression of *NOS2* induced by *S. epidermidis* was suppressed by 66.7-fold in the presence of the inhibitors. Likewise, transcription of *IL1A, NFkB* and *IRGM* was suppressed, respectively, by: 183-fold; 5.9-fold and 15-fold. Pre-incubation of the cells with 5-AzaC reduced the transcriptional activation of *ATG5*, *ATG8, PARK2* and *NOS2* in the challenged BMSCs in 6-, 4.3-, 3.6- and 64-fold respectively.

**Figure 2:**
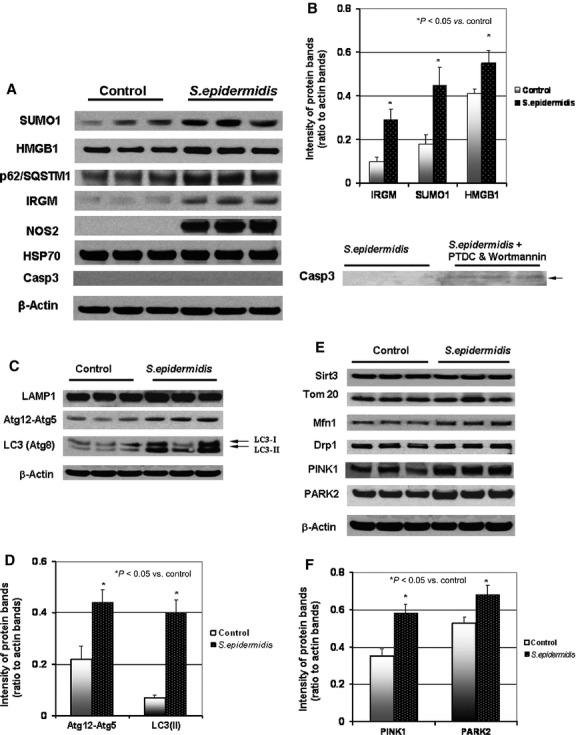
Immunoblot analysis of autophagy and stress-response proteins in BMSCs challenged with *Staphylococcus epidermidis*. The protein extracts were obtained from the BMSC cultures 24 hrs after a challenge with *S. epidermidis,* except the cells pre-treated with 10 μM PDTC and 200 nM Wortmannin (see Materials and methods) were collected at 5 hrs post-challenge. (A) Immunoblot bands of stress-responding proteins. (B) Densitometrical analysis of the protein bands shown in A; the displayed data are ratio of the protein band densities to the densities of the respective actin bands. (C) Immunoblot bands of autolysosomal proteins. (D) Densitometrical analysis of Atg12-Atg5 and LC3(II) bands shown in C; the displayed data are ratio of the protein band densities to the densities of the respective actin bands. (E) Immunoblot bands of mitochondrial and mitophagy-related proteins. (F) Densitometrical analysis of PINK1 and PARK2 bands shown in E; the displayed data are ratio of the protein band densities to the densities of the respective actin bands.

**Figure 3:**
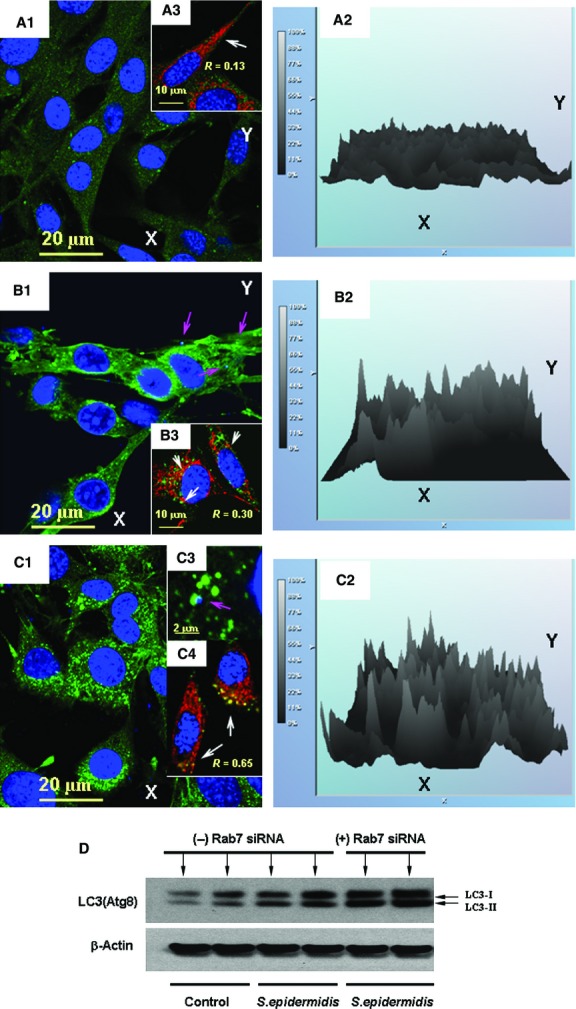
Spatial alterations in autophagosomal LC3 in the presence of microtubule-interfering agents. Assessment of autophagic flux in BMSCs challenged with *Staphylococcus epidermidis*. Analysis of immunoreactivity of LC3 protein in BMSC cells is shown in A–D. BMSC mitochondria were visualized with immunofluorescence of Tom20, a mitochondrial marker. The images shown in the figure are representative ones of analyses of three specimens obtained from three experiments. (A1) LC3-projections (green channel) in control BMSCs. (B1) LC3-projections (green channel) in BMSCs fixed at 24 hrs following the bacterial challenge. (C1) Same as (B1), but BMSCs were incubated with 50 μM vinblastine for 6 hrs before fixation. Accumulation of LC3-positive puncta appeared in images displayed in B1 and C1. (A2–C2) Relative intensities of the LC3-immunofluorescence projections presented in the respective panels (A1–C1) Projection of the nuclear DNA appeared in blue (counterstaining of nuclei with Hoechst 33342) in A1, A3, B1, B3, C1, C3 and C4. Spatial interactions of bacterial DNA with LC3-positive puncta appears in turquoise as result of interference of green and blue colours (indicated with pink arrows in B1 and C3). Projections of Tom20 (red channel) and LC3 (green channel) are displayed in panels A3, B3 and C4. Co-localization of LC3-positive puncta with Tom20 appeared in yellow (as result of interference of green and red) and is indicated with white arrows in B3 and C4. The confocal images were taken with pinhole setup to obtain 0.3 μm Z-sections. For assessment of the amounts of LC3-positive puncta, only puncta with diameter ≥0.2 μm were counted. (D) Immunoblot assessment of effect of Rab7 siRNA on amount of LC3I/LC3II in BMSCs challenged with *S. epidermidis*. The cells were treated with lipofectamine in the absence (-) or presence (+) of 10 nM Rab7 siRNA overnight, (http://tools.lifetechnologies.com/content/sfs/manuals/cms_084833.pdf). The protein extracts were obtained from the BMSC cultures 24 hrs after a challenge with *S. epidermidis*.

The data on gene transcriptional activation in the challenged BMSCs were consistent with the observed up-regulation of the respective proteins analysed by immunoblotting. Substantial increases in levels of the stress-response proteins (IRGM, SQSTM1/p62, iNOS, HMGB1, SUMO1), autolysosomal proteins (Atg12-Atg5, MAP/LC3(Atg8), and mitophagy-related proteins (PINK1 and PARK2) were observed at 24 hrs following bacterial challenge (Fig.2A–F). Moreover, the balance between LC3-I and LC3-II isoforms was dramatically shifted towards the lipidated isoform, LC3-II (Fig.2C). Indeed, the ratio of immunoblot bands of LC3-II and LC3-I (*i.e*. LC3-II/LC3-I) in control cells was 0.45 ± 0.05 a.u. compared to 1.57 ± 0.32 a.u. in the challenged cells (*P* < 0.03; *n* = 3; Fig.2C). This effect can be associated with increases in the Atg4-dependent conversion of LC3-I to LC3-II, which is essential for the autophagosome formation 28. Therefore, accumulation of LC3-II in BMSCs could likely reflect up-regulation of autophagy following the observed bacterial phagocytosis. However, as the cell steady-state balance of LC3-II *per se* does not reflect the dynamics of the autophagy flux, the efficacy of autolysosomal events can also affect the LC3-II levels (note, as shown in Fig.3C, BMSCs exhibit a high expression of LAMP1, a marker of lysosomes). Indeed, when LC3-I is converted to LC3-II, the latter binds to the expanding phagophore/auophagosome and remains within autophagosomes even after fusion with lysosomes. Then, LC3-II can be either delipidated and recycled or degraded by hydrolytic enzymes in the autolysosome 29. At this stage, inhibition and/or impairment of the auophagosomal/lysosomal fusion can result in accumulation of autophagosomes in cells and raise the amount of LC3-II protein. Therefore, for clarification of LC3 dynamics, we further (below) conducted monitoring of autophagic flux during auophagosomal/lysosomal fusion.

Interestingly, the BMSC response to the bacterial challenge caused a drastic increase in expression of iNOS (Table2 and Fig.2), which produces nitric oxide and thus, mediates microbiostatic defence mechanism. However, the side effect of nitric oxide production in the cells was nitroxidative stress to the cellular constituents (shown below). Evidently, the stress-adaptive mechanisms expressed under the above experimental conditions may allow the cells to avoid ‘self-inflicted’ oxidative injury and activation of apoptosis as recently reported 39. In this light, the presence of high levels of constitutively expressed HSP70, which can suppress the processing of caspase 3 (Casp-3, a marker of apoptosis), would explain a failure to detect activated Casp-3 protein in the challenged cells (Fig.2).

### Autolysosomal Activity in BMSCs Following the Challenge with *S. epidermidis*

The levels of LC3-II (measured by immunoblotting) and LC3-positive puncta (assessed by fluorescent imaging) are commonly used for monitoring of autophagosomal activity. Ultimately, the mature autophagosomes carrying cargos (*e.g*. bacteria or impaired organelles) for lysosomal destruction have to be fused with lysosomes to form autolysosomes. The autophagosomal–lysosomal fusion and lysis of the microorganisms are shown in TEM micrographs of BMSCs (Fig.1). This fusion step is a part of autophagic flux associated with autolysosomal activity. In contrast, inhibition of fusion of matured autophagosomes with lysosomes results in accumulation of LC3-positive puncta that, in turn, can be used as an index of autolysosomal activity 35.

As shown in Figure3A1 and B1, the bacterial challenge resulted in a significant increase in the amount of LC3-positive punctate foci in the cells; that is from 9 ± 3 puncta/cell (control) to 34 ± 11 puncta/cell (challenge); *P* < 0.01, *n* = 20). This effect was accompanied by an increase in LC3-related immunofluorescence in the challenged cells (Fig.3A2 and B2). As shown in Figure3B1, the phagocytized bacteria (identified by fluorescence of the stained DNA) were encapsulated into LC3-positive vesicles. Increases in spatial co-localization of LC3 with mitochondrial Tom20 is shown in Figure3B3, *i.e. R* = 0.30 compared to *R* = 0.13 in control. The steady-state balance of LC3-positive puncta was drastically affected in the presence of vinblastine, a microtubule depolymerizing agent that induces the accumulation of autophagic vacuoles by preventing their lysosomal fusion and degradation. Indeed, the effect of vinblastine was associated with a twofold increase in amounts of LC3-positive puncta in the challenged cells (*i.e*. 74 ± 12 puncta/cell; *P* < 0.01, *n* = 20; compared to those without the inhibitor, *i.e*. 34 ± 11 puncta/cell) (Fig.3B1, C1, B2, C2). Moreover, as shown in Figure3, the LC3-positive puncta was co-localized with the bacterial DNA (C3) and mitochondrial Tom20 (C4), *i.e. R* = 0.65 compared to *R* = 0.13 with control Tom20 (A3). Overall, the above data indicate the presence of autophagic flux in the bacteria-challenged cells.

It has been well documented that numerous molecular components regulate autophagic/lysosomal fusion. For example, it has been shown recently that Rab 7, the small GTPase associated with vesicular membranes, is essential for autolysosomal progression and autophagic flux 40. To interfere with the Rab7-mediated autolysosomal pathway, we transfected BMSCs with Rab7 siRNA to suppress the *RAB7* gene. The effect of this interference was assessed with immunoblotting of LC3 protein in the cells. As shown in Figure3D, interference with Rab7-mediated events resulted in a substantial accumulation of LC3I/LC3II in the bacteria-challenged cells.

### Remodelling of the Mitochondrial Network in BMSCs Challenged with *S. epidermidis*

**Figure 4:**
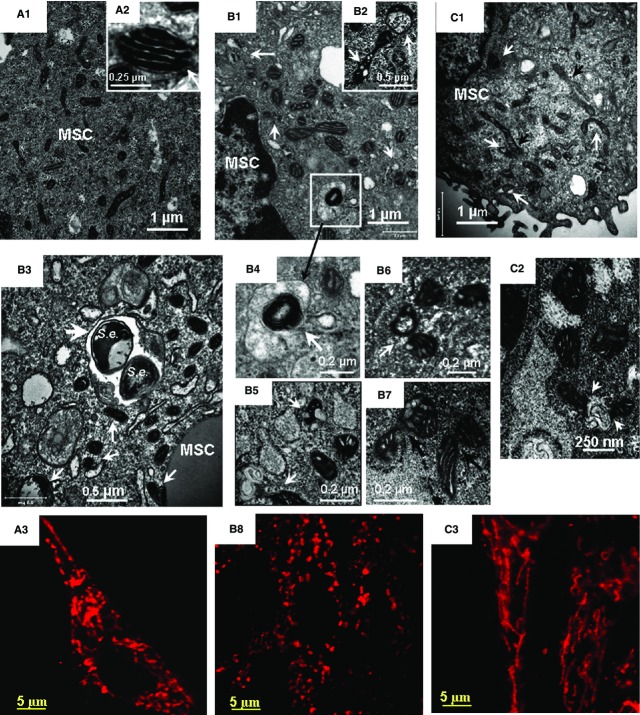
Transmission electron microscopy (TEM) and confocal immunofluorescence imaging of mitochondrial remodelling in BMSCs challenged with *Staphylococcus epidermidis*. TEM micrographs and projections of Tom 20 obtained from control BMSC (A) and BMSCs fixed at 5 hrs (B) and 24 hrs (C) following the bacterial challenge. The images shown in the figure are representative ones of analyses of three specimens obtained from three experiments. (A1, B1, B3 and C1) Low-magnification TEM micrographs of BMSCs. (A2) High-magnification TEM micrograph of normal mitochondrion. (B1) Aberrant mitochondria and formation of mitophagosome are indicated with white arrows. (B2) High-magnification TEM micrograph of a swollen (aberrant) mitochondrion with lysis of cristae and transfer of an aberrant mitochondrion to autophagosome (indicated with white arrows). (B3) Co-localization of xenophagy of *S. epidermidis* (*S.e*.) and mitophagy events in BMSC. Aberrant mitochondria and formation of mitophagosome are indicated with white arrows, *S. epidermidis* in an autolysosome is shown with a white arrowhead. (B4) (ROI indicated in B1). Image of aberrant mitochondria in autolysosome. (B5 and B6) Autophagy of aberrant mitochondria (mitophagosome formation). (B7) Remodelling of aberrant mitochondria as indicated with black arrows. (C1 and C2) TEM images of mitochondrial alterations observed 24 hrs after bacterial challenge. Formation of fragmented aberrant mitochondria (white arrows) and elongated mitochondrial tubules (black arrows) is shown in C1. Fusions of aberrant mitochondria with autophagosomes are indicated in C2 with white arrows. (A3, B8 and C3) Confocal immunofluorescence images of mitochondrial remodelling in BMSCs. The mitochondrial networks were visualized using projections of Tom20 (red channel), a mitochondrial marker. (A3) Control BMSCs. The mitochondrial network is presented by a combination of elongated and round-shape projections. (B8) Mitochondrial alterations observed 5 hrs after bacterial challenge. The mitochondrial network is comprised of numerous round-shape projections. (C3) Mitochondrial alterations observed 24 hrs after bacterial challenge. The mitochondrial network appeared in a form of reticular long-length structures formed because of mitochondrial fusion. TEM conditions: Same as indicated in Figure2. The confocal images were taken with pinhole setup to obtain 0.5 μm Z-sections.

Bone marrow mesenchymal stromal cells challenged with *S. epidermidis* resulted in dramatic alterations in the mitochondrial network that was analysed with TEM and immunofluorescence confocal imaging of mitochondrial protein Tom20 (translocase of outer mitochondrial membrane subunit 20). TEM micrographs and immunofluorescence projections of Tom 20 in control and challenged cells are shown in Figure4. TEM imaging shows that the mitochondrial network in control BMSCs was represented by a combination of round and elongated organelles containing discrete cristae of high density (Fig.4A1 and A2). Similar observations were obtained with fluorescence microscopy (Fig.4A3). It should be noted that, overall, approximately 4% of the organelles appeared aberrant in the non-challenged cells. Extensive mitochondrial swelling and cristae fragmentation was observed at 5 hrs post-challenge; approximately 20% organelles appeared aberrant at this stage (Fig.4B1, B2 and B3). The consequences of the mitochondrial injury can include either activation of CytC/Casp-3-dependent apoptosis or pro-survival autolysosomal mechanisms for recycling of the cellular constituents. As determined, the challenged BMSCs employed the autolysosomal mechanism for degradation of the aberrant mitochondria (Fig.4B1–B7). Note, co-localization of xenophagy of *S. epidermidis* (*S.e*.) and mitophagy events in BMSC is shown in Figure4B3.

The results of immunofluorescence image analysis suggested that the cell stress response to the bacterial challenge was accompanied by a substantial mitochondrial fragmentation (Fig.4B3 and B8). Dynamic remodelling of the mitochondrial network was observed over 24 hrs post-challenge (Fig.4C1 and C2). As a result, almost the entire ‘mitochondrial body’ was represented by reticular structures at this time-point (Fig. 4C3). These mitochondrial re-arrangements were not likely accompanied by alteration in the cell mitochondrial mass as the results of the Western blot analysis did not reveal a significant increase in Tom20 and Sirt3 mitochondrial proteins (Fig.2). A slight elevation in the amount of Mfn1 protein in the challenged cells (compared to control) could reflect increased transcriptional activation of *Mfn1* (Table.3).

**Figure 5:**
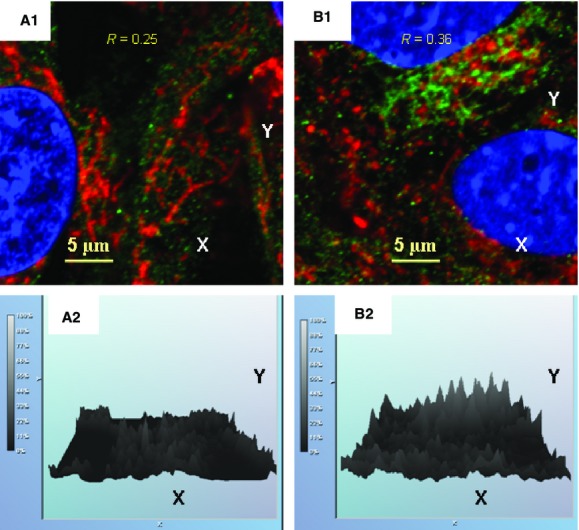
Immunofluorescence confocal image analysis of spatial localization of IRGM in BMSCs challenged with *Staphylococcus epidermidis*. (A1) Control BMSCs. Mitochondrial network was visualized by projections of Tom20 (red channel), a mitochondrial marker, projections of IRGM protein appear in green. Projections of the nuclear DNA appear in blue (counterstaining of nuclei with Hoechst 33342). Index of spatial correlation of IRGM and Tom20 was defined as *R* = 0.25 (see Materials and methods). (B1) Alterations in mitochondrial translocation of IRGM (green channel) observed 24 hrs after bacterial challenge. Mitochondrial network was visualized by projections of Tom20 (red channel). Index of spatial correlation of IRGM and Tom20 is defined as *R* = 0.36 (see Materials and methods). (A2 and B2) Relative intensities of the IRGM-immunofluorescence projections presented in the respective A1 and B1. Note: Spatial co-localization of IRGM and Tom20 appears in yellow as result of interference of green and red colours. The confocal images were taken with pinhole setup to obtain 0.3 μm Z-sections. The images shown in the figure are representative ones of analyses of three specimens obtained from three experiments.

There are numerous potential mechanisms that could initiate and drive the inflammagen-induced remodelling of the mitochondrial network. It has been reported recently that IRGM can translocate to mitochondria where it regulates autophagy in association with mitochondrial fission 28. In these events, IRGM can bind to mitochondrial cardiolipin and consequently induce mitochondrial depolarization 28 that, in turn, can promote mitochondrial recruitment of PARK2 24–26. Taking into consideration that the challenge of BMSCs with *S. epidermidis* resulted in substantial increases in expression of IRGM (Fig.2; Table2), we further analysed spatial distribution of IRGM in the challenged cells in association with a mitochondrial marker Tom20. The results of confocal immunofluorescence image analyses presented in Figure5 were corroborated with immunoblotting data (Fig.2). Moreover, these analyses confirmed the presence of notable co-localization of IRGM with mitochondria in the bacteria-challenged cells (*R* = 0.36) (Fig.5B).

**Figure 6:**
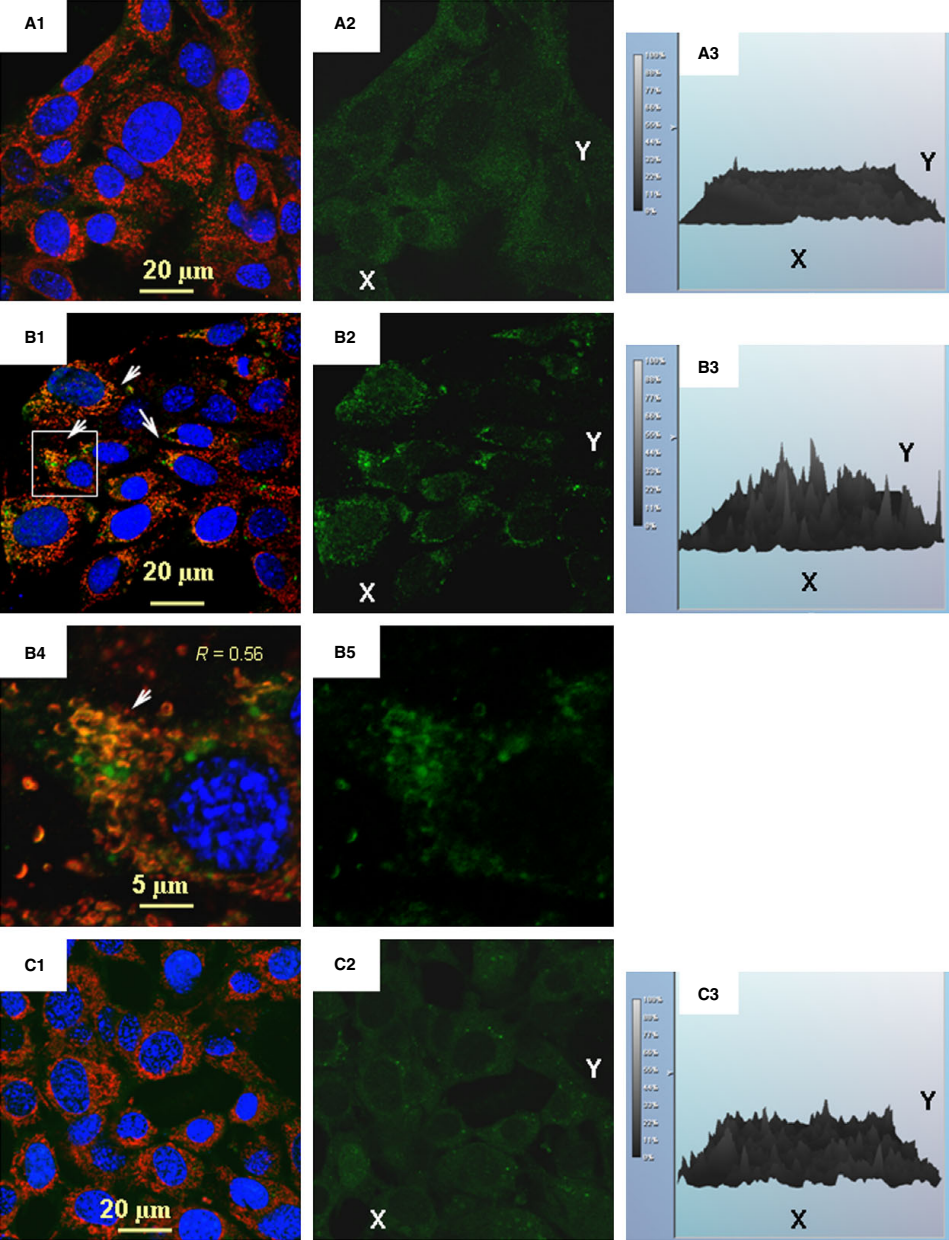
Immunofluorescence confocal image analysis of 3-nitrotyrosine (3-NTyr) in mitochondrial proteins in BMSCs challenged with *S. epidermidis*. The mitochondrial network was visualized by projections of Tom20 (red channel), a mitochondrial marker (projections of 3-NTyr appear in green). Projections of the nuclear DNA are displayed in blue (counterstaining of nuclei with Hoechst 33342). Spatial co-localization of 3-NTyr and Tom20 appears in yellow as result of interference of green and red colours. The confocal images were taken with pinhole setup to obtain 0.3 μm Z-sections. The images shown in the figure are representative ones of analyses of three specimens obtained from three experiments. (A1, B1 and C1) Projections of Tom20 and 3-NTyr in control BMSCs (A1), BMSCs challenged with *S. epidermidis* (B1), and BMSCs challenged with *S. epidermidis* in the presence of 100 μM LNIL, an inhibitor of iNOS (C1). (B4) Magnification of the selected AOI is indicated with a square in B1. (A2, B2, C2 and B5) Immunofluorescence of 3-NTyr in the images shown in A1, B1, C1 and B4 respectively. (A3, B3 and C3) Histograms representing relative intensities of immunofluorescence of 3-NTyr shown in A2, B2 and C2 respectively.

The multifactorial response of BMSCs to the bacterial challenge included a drastic increase in expression of iNOS (see data above), which can produce a massive amount of nitric oxide. The released nitric oxide is known to cause nitrosative stress and promote mitochondrial fission, which is associated with the release of superoxide 15,16,36. Under these conditions, one would expect the formation of peroxynitrite from nitric oxide and superoxide, and the subsequent peroxynitrite-mediated nitroxidation of tyrosine in mitochondrial proteins to form 3-nitrotyrosine (3-NTyr). Indeed, immunofluorescence analysis of BMSCs showed a remarkable increase in 3-NTyr immunoreactivity co-localized with mitochondria that occurred after the challenge with *S. epidermidis* (Fig.6). It should be noted that 3-Ntyr-positive organelles were round-shaped and the mitochondrial reticulum was fragmented (Fig.6B4).

Overall, both IRGM and oxidative injury could, at least in part, contribute to the mechanisms driving mitochondrial remodelling upon development of the BMSC stress response.

### Dynamics of Drp1 and PARK2 Proteins in BMSCs Challenged with *S. epidermidis*

It is well documented that mitochondrial fission and the PINK1–PARK2-mediated mitophagy require recruitment of Drp1 and PARK2 proteins to the targeted mitochondria; characteristic images of these events have been presented in numerous reports 18–25. Drp1 translocation to mitochondria is accompanied by assembly of circular oligomers involving Drp1 attached to the outer membrane-anchored mitochondrial fission factor protein followed by excision of the mitochondrial tubules perpendicular to the cylinder axis; this effect is shown in Figure4B6. Binding of the activated mitochondrial kinase PINK1 to the Tom20 complex and PINK1-mediated phosphorylation of the PARK2 ubiquitin-like domain trigger translocation of PARK2 from cytosol to mitochondria, thereby priming mitophagy in the depolarized mitochondria 12,25,31.

**Figure 7:**
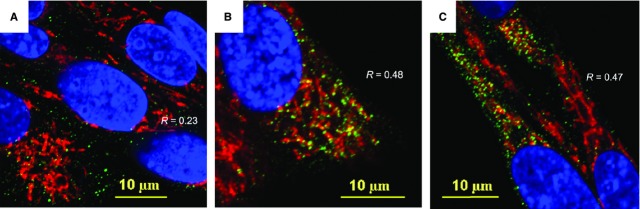
Immunofluorescence confocal image analysis of mitochondrial translocation of Drp1 fission protein in BMSCs challenged with *Staphylococcus epidermidis*. (A) Control BMSCs. The mitochondrial network was visualized using projections of Tom20 (red channel), a mitochondrial marker. Projections of Drp1 protein appear in green. Projections of the nuclear DNA appear in blue (counterstaining of nuclei with Hoechst 33342). Index of spatial correlation of Tom20 and Drp1 is defined as *R* = 0.23 (see Materials and methods). (B) Alterations in mitochondrial translocation of Drp1 (green channel) observed 5 hrs after bacterial challenge. The mitochondrial network is displayed as projections of Tom20 (red channel). Index of spatial correlation of Tom20 and Drp1 is defined as *R* = 0.48 (see Materials and methods). (C) Alterations in mitochondrial translocation of Drp1 (green channel) observed 24 hrs after bacterial challenge. The mitochondrial network appears as long-length thread-like projections of Tom20 (red channel). Index of spatial correlation of Tom20 and Drp1 is defined as *R* = 0.47. The presence of interaction between Drp1 and Tom20 appears in yellow as result of interference of red and green colours. The confocal images were taken with pinhole setup to obtain 0.3 μm Z-sections. The images shown in the figure are representative ones of analyses of three specimens obtained from three experiments.

**Figure 8:**
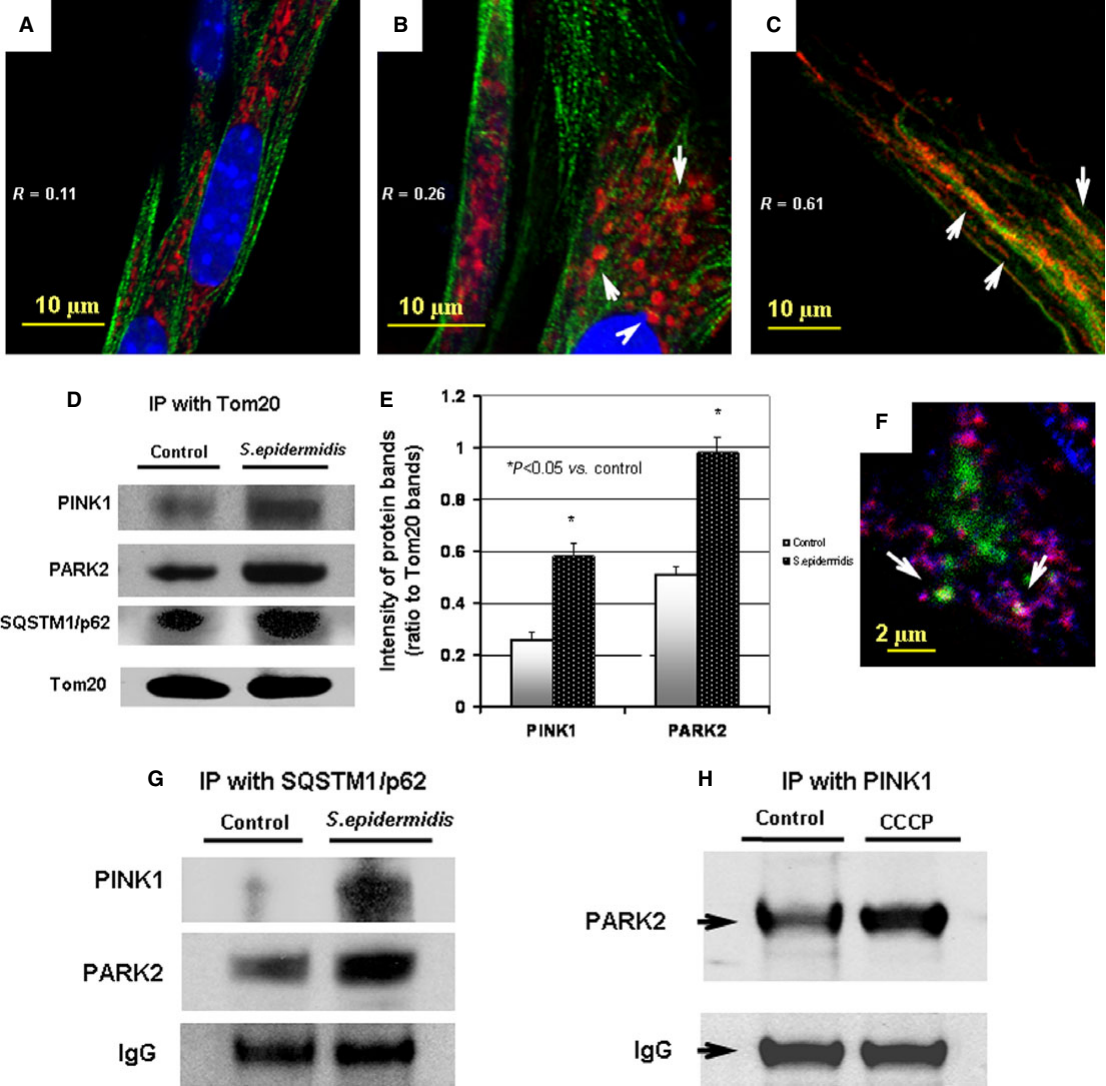
Immunofluorescence confocal image analysis of mitochondrial translocation of PARK2 protein in BMSCs challenged with *Staphylococcus epidermidis*. The mitochondrial network was visualized with projections of Tom20 (red channel), a mitochondrial marker. Projections of PARK2 protein appear in green in A, B, C and in blue in F. Projection of the nuclear DNA appears in blue (counterstaining of nuclei with Hoechst 33342) in A, B, C. Spatial co-localization of Tom20 and PARK2 appears in yellow in A, B, C as result of interference of red and green colours. Spatial co-localization of Tom20 and PARK2 appears in pink colour in F as result of interference of red and blue colours. (A) Control BMSCs. Index of spatial correlation of Tom20 and PARK2 was determined as *R* = 0.11 (see Materials and methods). (B) Alterations in mitochondrial translocation of PARK2 (green channel) observed 5 hrs after bacterial challenge. The mitochondrial network appears as numerous round-shape projections of Tom20 (red channel). Index of spatial correlation of Tom20 and PARK2 determined as *R* = 0.26 (see Materials and methods). (C) Alterations in mitochondrial translocation of PARK2 (green channel) observed 24 hrs after bacterial challenge. The mitochondrial network appears as long-length thread-like projections of Tom20 (red channel). Index of spatial correlation of Tom20 and PARK2 was determined as *R* = 0.61. The confocal images were taken with pinhole setup to obtain 0.3 μm Z-sections. The images shown in the figure are representative ones of analyses of three specimens obtained from three experiments. (D) Immunoblotting analysis of Pink1, PARK2 and proteins co-immunoprecipitated with Tom20 protein. Immunoprecipitation (IP) assessment of the protein interaction on the BMSC mitochondria 24 hrs after bacterial challenge. (E) Densitometrical analysis of the protein bands shown in D; the displayed data are ratio of the protein band densities to the densities of the respective Tom20 bands. (F) Immunofluorescence assessment of spatial interaction of PARK2 (blue channel) and LC3 (green channel) with mitochondrial Tom20 (red channel) in BMSC subjected the bacterial challenge (24 hrs). Co-localization of Tom20 with PARK2 and LC3 appears in form of white circles, *i.e*. mitophagosomes (indicated with white arrows) as result of interference of blue, green and red colours. (G) Immunoblotting analysis of PINK1 and PARK2 proteins co-immunoprecipitated with SQSTM1/p62 protein. IP assessment of the protein interaction on the BMSC mitochondria 24 hrs after bacterial challenge. (H) Immunoblotting analysis of PARK2 protein co-immunoprecipitated with mitochondrial Pink1protein. IP-assessment of increase in interaction of Pink1 with PARK2 in the BMSC mitochondria after mitochondrial depolarization by treatment with 10 μM CCCP, a mitochondrial uncoupler, for 6 hrs.

The results of immunofluorescence confocal imaging of Drp1, PARK2 and Tom20 are shown in Figures7 and 8. Analysis of the projections of Tom20 and Drp1 indicates that, indeed, the bacterial challenge to BMSCs was accompanied by increased translocation of Drp1 to mitochondria (Fig.7). Thus, while the spatial correlation of Drp1 and Tom20 in control cells was *R* = 0.23 (Fig.7A), the R-values increased to 0.48 and 0.47, 5 and 24 hrs after challenge respectively (Figure7B and C). A similar effect was observed in the dynamics of PARK2 protein Fig.8). Analysis of the projections of Tom20 and PARK2 indicated that in control BMSCs, the spatial correlation of PARK2 and Tom20 was *R* = 0.11; and PARK2 protein was predominantly bound to the cytoskeletal filaments (Fig.8A) in a manner similar to the observation reported recently by Huynh *et al*. 32. A response to the stress induced by the bacterial challenge was associated with PARK2 translocation from the cytoskeletal filaments to mitochondria increasing the spatial correlation between PARK2 and Tom20 proteins, 5 hrs (*R* = 0.26) and 24 hrs (*R* = 0.61) post-treatment (Fig.8B and C).

The results of immunofluorescence analysis of Drp1 and PARK2 are corroborated with the immunoblotting data presented and discussed above (Fig.2E). Indeed, Drp1 and PARK2 are constitutively expressed in BMSCs; therefore, they could be promptly recruited to mitochondria upon respective stimuli. The increase in PARK2 presence at mitochondria was confirmed using immunoprecipitation analysis of complexes of the mitochondria-specific proteins Tom20 and PINK1 with PARK2 (Fig.8D–F). Interestingly, this effect was also accompanied by (*i*) the appearance of co-localization of LC3-positive vesicles with PARK2-positive mitochondria (Fig.8F) and (*ii*) increases in the mitochondrial presence of SQSTM1/p62 (Fig.8D) and SQSTM1/p62 co-precipitation with mitochondrial PINK1 and PARK2 (Fig.8G). It was noted that the amount of the autophagic adapter protein SQSTM1/p62 was slightly increased in the challenged cells (Fig.2). Thus, the arbitrary density of immunoblot bands of SQSTM1/p62 (normalized to actin; Fig.2) in the challenged cells was 0.35 ± 0.02 a.u., while the same parameter in the control cells was 0.24 ± 0.01 a.u. (*P* < 0.03, *n* = 3).

Overall, the above observations implicated the PINK1/PARK2 mechanism in the observed remodelling of the BMSC mitochondrial network following the bacterial challenge.

## Discussion

Extracellular and intracellular antibacterial mechanisms constitute complex lines of the host–cell defence barriers that sustain immune homoeostasis of multicellular organisms 6,30,33. Various cell lineages employ autophagy for the terminal elimination of phagocytized microorganisms, and, in many cases, this autophagy route is crucial for the host resistance to bacterial translocation and propagation 6,27,30. With consideration of the above, we assumed that assessment of the autophagy/autolysosomal pathway in challenged BMSCs could shed light on the role of the ubiquitous mesenchymal stromal cells in the barrier response mechanisms implemented against invading *S. epidermidis*.

*Staphylococcus epidermidis* is one of the most common microorganisms colonizing the epithelial surfaces 36,41. Impairment of tissue barriers frequently causes chronic infections with *S. epidermidis,* which demonstrates a pronounced capacity to evade host defences and to inactivate white blood cells. Thereby, bacteraemia because of *S. epidermidis* can cause long-term effects on immune tissues including bone marrow, while sepsis becomes a potentially fatal issue 36–38,41.

Evidently, the challenge of BMSCs with bacteria produced the inflammagenic stress mediated by the PRE/DAMP response elements and was accompanied by metabolic stress. It has been proposed recently, that a pattern of these events, can be associated with reconstitution and remodelling of the entire ER?mitochondrial network and provoke mitochondrial dysfunction 6,14,15,35. This assumption is corroborated by numerous observations indicating that activation of IRGM, ER stress, or redox stress can trigger mitochondrial membrane depolarization, permeabilization, and swelling, and thus promote mitochondria-dependent apoptotic cell death 15,17,35,42. All of the above make mitochondria crucial players in the current paradigm for cellular homoeostasis and survival 16,17,35,42,43.

Mitochondria-driven cell death is one of the most common innate defence mechanisms against invading pathogens. However, some microorganisms can subvert these death mechanisms and maintain viability of the host cells for the ultimate enhancement of their replication and propagation 44. Translating these scenarios to bacteraemia, the affected cells may potentiate septicaemia caused by the bacterial inflammogens*,* because of the ability of impaired mitochondria released by affected cells to operate as paracrine mediators of danger or inflammation 17,43 unless the impaired organelles are destroyed with autolysosomes.

Recent time-lapse microscopy data indicate that mitochondria are extremely dynamic organelles; they constantly undergo fusion and fission processes, which regulate architecture and efficiency of mitochondrial tubular networks as well as excision of compromised mitochondrial sections 7–11. These events are followed by removal and degradation of the aberrant organelles *via* mitophagy/autolysosomal mechanisms 10,12,13. We demonstrated that BMSCs constitutively express proteins which sustain signalling modules essential for activation and execution of mitochondrial remodelling and mitophagy. These proteins include (but are not limited to) Mfn1, Drp1, PINK1, PARK2, SUMO1, SQSTM1/p62 and LC3 proteins. The challenge of BMSCs with *S. epidermidis* resulted in (*i*) the formation of aberrant mitochondria in the cells, (*ii*) an increase in both expression of the mitophagy-mediated proteins (*e.g*. PARK2, SQSTM1/p62 and LC3) and their interaction with mitochondria and (*iii*) autophagosomal sequestration of aberrant mitochondria.

Overall, the BMSCs challenged with the *S. epidermidis* microorganisms experienced dramatic stress and mitochondrial injury. However, the cells neither became infected nor underwent apoptosis, but rather activated (*i*) stress-response adaptive mechanisms, (*ii*) antibacterial xenophagy, (*iii*) mitochondrial remodelling and (*iv*) mitophagy. In this respect, the subsequent autolysosomal recycling of the aberrant mitochondria could both suppress the mitochondria-dependent apoptosis and reduce the risk of release of mitochondria-originated stress factors. Therefore, we postulate that the observed up-regulation of mitophagy can be considered as a pro-survival homoeostatic mechanism implemented by the challenged BMSCs. From a biomedical perspective, the reported BMSC effects can contribute to the innate defence response of bone marrow stroma to the sepsis associated with radiation-induced injury and trauma 1,45.

## Disclaimer

The opinions or assertions contained herein are the authors private views and are not to be construed as official or reflecting the views of the Uniformed Services University of the Health Sciences, Armed Forces Radiobiology Research Institute, the U.S. Department of Defense, or the National Institutes of Health. The commercial products identified in this paper do not imply recommendation or endorsement by the federal government and do not imply that the products identified are necessarily the best available for the purpose.

## Supporting Information

**Figure S1** Bright-field microscopy of bone marrow stromal cell, i.e. CFU-F, culture; clonal cell phenotyping; and assessment of the cell monolayers challenged with *S. epidermidis*.
