# Supplementary figures and images for: Autophagy and mitochondrial remodelling in mouse mesenchymal stromal cells challenged with Staphylococcus epidermidis
Source: J Cell Mol Med. 2015 Feb 26;19(5):1133–50. doi: 10.1111/jcmm.12518 (PMC4420615; doi:10.1111/jcmm.12518)

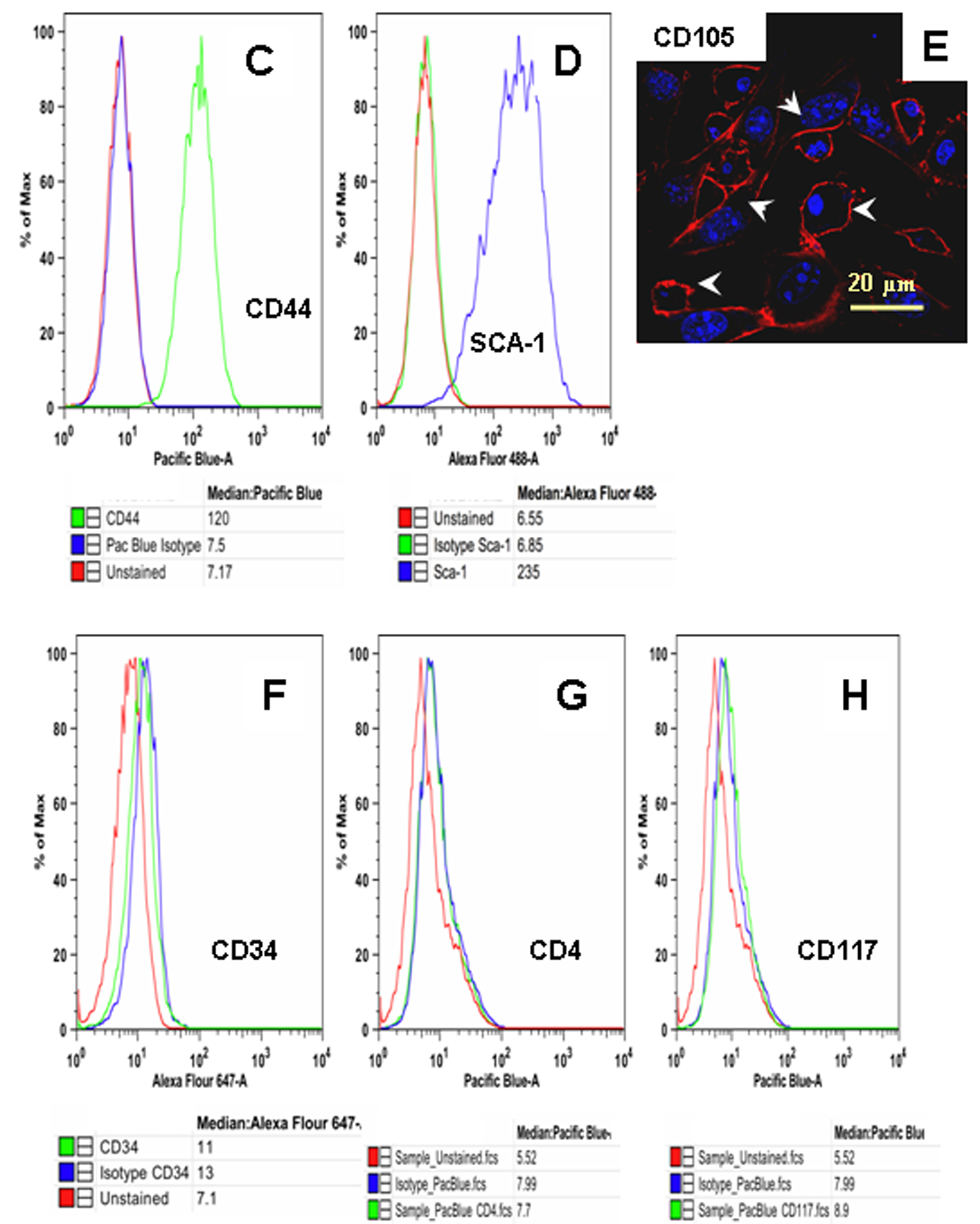

Supplement: Supplementary file 1 [file jcmm0019-1133-sd1.tif]

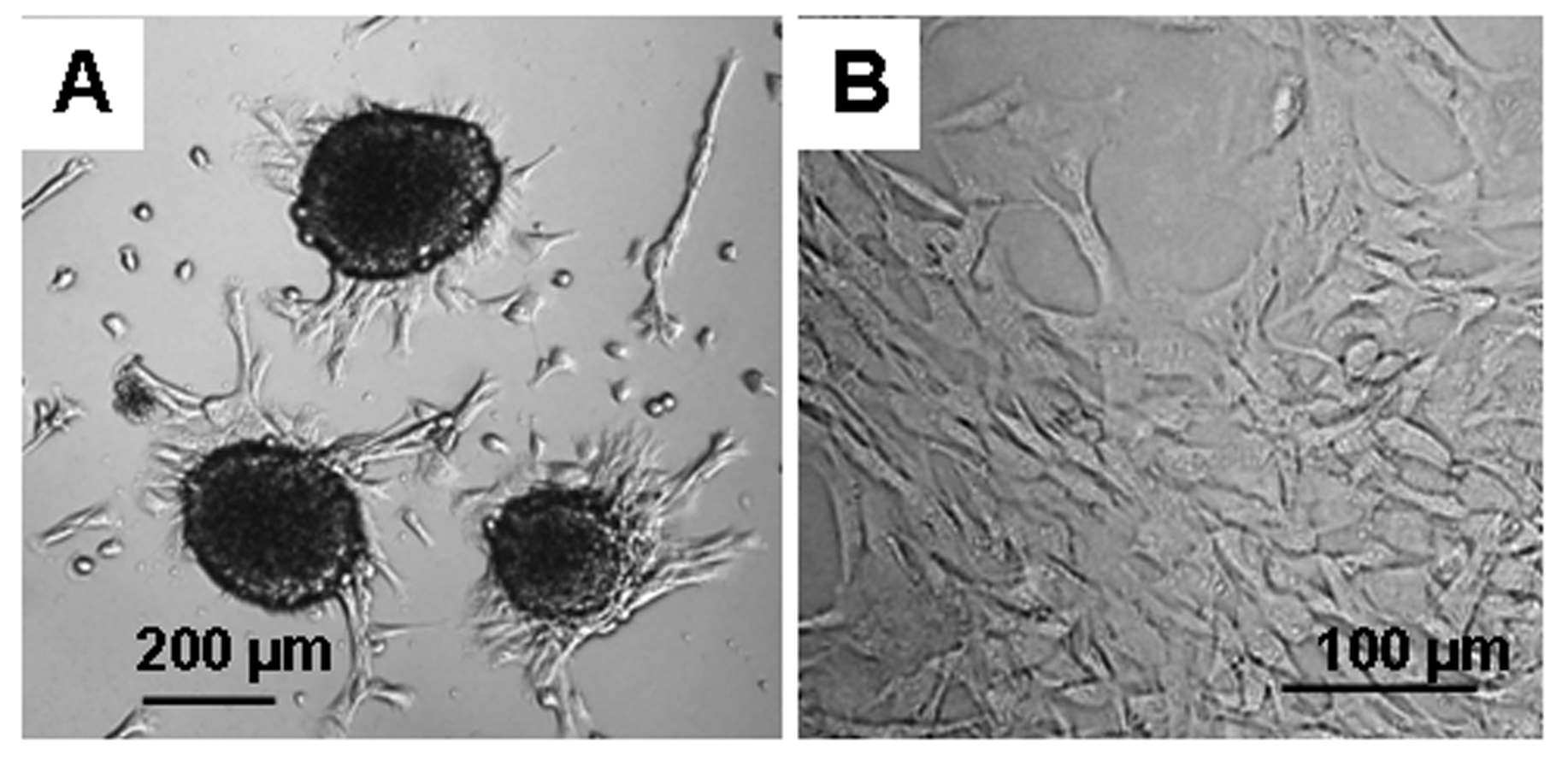

Supplement: Supplementary file 2 [file jcmm0019-1133-sd2.tif]

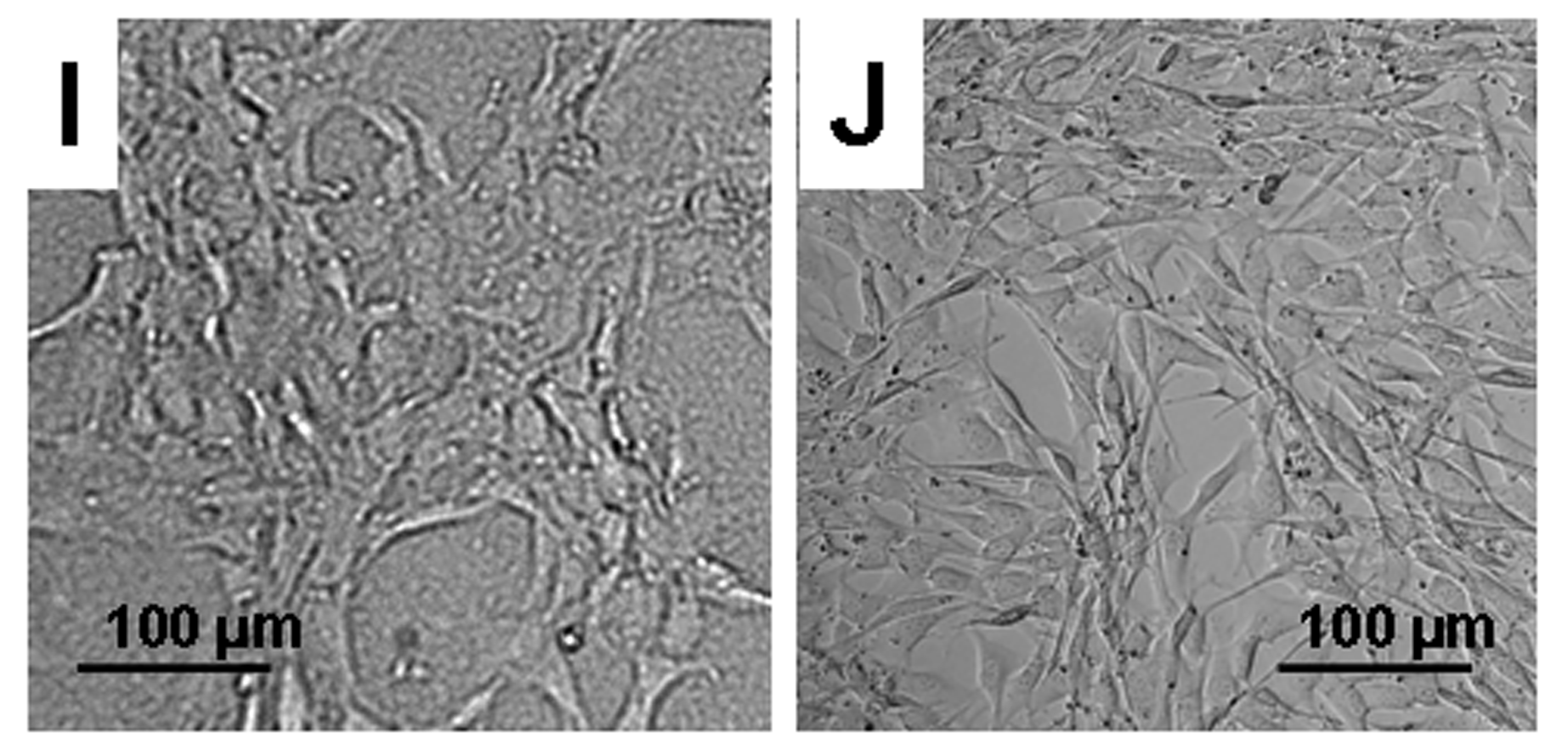

Supplement: Supplementary file 3 [file jcmm0019-1133-sd3.tif]
